# Inpatient detoxification for alcohol and other drug use: qualitative study of patients’ accounts of their relationships with staff

**DOI:** 10.1186/s13722-024-00523-0

**Published:** 2024-12-02

**Authors:** Joanne Neale, Beth Cairns, Kevin Gardiner, Wulf Livingston, Trevor McCarthy, Andrew Perkins

**Affiliations:** 1 Institute of Psychiatry, Psychology & Neuroscience, National Addiction Centre, King’s College London, London, SE5 8BB UK; 2 Figure 8 Consultancy Services Ltd, Dundee, UK; 3 Faculty of Social and Life Sciences, Wrexham University, Wrexham, UK

**Keywords:** Detoxification, Inpatient, Substance use, Therapeutic alliance, Qualitative research

## Abstract

**Background:**

The therapeutic alliance is an important predictor of treatment outcomes but people who use alcohol and other drugs report mixed views of treatment providers. We analysed patients’ accounts of inpatient detoxification staff to ascertain whether, and if so how, relationships with them, and thus the therapeutic alliance, might be improved.

**Methods:**

Semi-structured qualitative interviews were conducted (in 2022/2023) with 20 people (14 males; 6 females) who had just completed inpatient detoxification in sixteen different facilities. Interviews were part of a larger longitudinal qualitative evaluation of an initiative to increase inpatient detoxification capacity across England.

**Results:**

Patients described how treatment was delivered by professionals with diverse roles. They rated staff highly and appreciated their personal qualities; the standard of medical care and non-medical services they provided; their willingness to provide privacy, freedom and choice; the support given at key points in the treatment journey; and the positive impact staff relationships had on their substance use and lives more generally. Criticisms of staff were infrequent, mostly related to specific individuals or events, and potentially more common when detoxification occurred within general hospitals rather than within specialist services.

**Conclusions:**

Patients’ accounts of staff in this study were more positive than documented in previous literature. However, the characteristics that patients appreciated (and disliked) were consistent with earlier research. There was scope to improve in some services and patient groups not interviewed may have held more negative views of staff. Overall, the holistic and patient-centred approach that staff adopted, and patients valued, appears to contribute to a good therapeutic alliance.

## Background

The quality of the relationship between healthcare professional and patient, often referred to as the therapeutic alliance, is a predictor of treatment outcomes for a range of mental health conditions [1–4]. For example, there is good evidence that a strong therapeutic alliance is associated with positive outcomes, particularly engagement and retention, in the treatment of alcohol and other drug (AOD) use [4–6]. Although factors affecting the quality of the relationship between people who use substances and those who treat them are not well understood, patient motivation, treatment readiness, and positive previous treatment experiences all appear to be linked to therapeutic alliance [4]. In contrast, patients’ demographic or diagnostic pre-treatment characteristics and therapist age and gender appear less predictive of good relationships [4].

Existing literature suggests that better patient outcomes are achieved by clinicians with more effective communication skills [7–10]. Relatedly, social workers who display warmth, empathy, acceptance, concern, and commitment, and who use authority and power appropriately, tend to establish more productive working relationships with people they support [11, 12]. When individuals who use alcohol and other drugs have been asked for their views on both generic and specialist addiction treatment providers, findings indicate that they appreciate professionals who are knowledgeable about AOD issues, have a positive and non-stigmatising attitude, encourage open and honest working relationships, are willing to listen, and are supportive, compassionate, friendly, and understanding [13–15].

Detoxification is a therapeutic procedure designed to manage people safely through the withdrawal symptoms associated with reducing or ceasing substance use [16–18]. It is generally intended to foster a person’s entry into long-term treatment and recovery [16, 17, 19] and, when undertaken in inpatient settings, tends to be medically managed with 24-hour support from a multidisciplinary team [18]. To date, there has been little research into how people undertaking inpatient detoxification evaluate staff working in those services. One exception is a study conducted in Florida, United States, which found that patients reported mixed negative and positive views. Thus, they appreciated staff who were kind, friendly, and helped them feel supported but were critical when staff conveyed judgement, were condescending, were new to the field, or lacked personal experience of substance use [8].

The scarcity of research exploring patients’ views of inpatient detoxification staff may relate to several factors. First, inpatient detoxification is less widely available than community-based treatments so there are comparatively fewer people accessing the treatment [20]. Secondly, inpatient detoxification is time limited (often 7–14 days), meaning that there may be less opportunity for a therapeutic alliance with staff to develop or for psycho-social interventions to occur. Thirdly, inpatient detoxification is frequently provided in a service that is geographically distant from patients’ homes, resulting in reduced opportunities for contact with staff before or after the treatment. Despite this, it has been argued that psychological support is an important part of the inpatient process [16, 18] and a primary goal of detoxification staff should be to build the therapeutic alliance so that patients are motivated to complete detoxification and subsequently enter outpatient treatment [16, 17, 21, 22].

In 2020, an independent review conducted in the United Kingdom (UK) found that there was significant under-provision of inpatient detoxification in England [23]. Responding to this, the UK Government announced an initiative to support the commissioning of inpatient detoxification services and increase capacity for those with complex needs. Grants were awarded to fifteen regional and sub-regional English consortia to commission additional bedspaces in a range of settings, with medically managed inpatient detoxification being the priority. Treatment was provided in new and existing facilities, including specialist detoxification services (or units) and specialist beds and wards in general hospitals. This paper is based on data collected as part of a wider qualitative evaluation of that initiative. The aim of the paper is to analyse patients’ accounts of inpatient detoxification staff to ascertain whether, and if so how, relationships with them, and thus the therapeutic alliance, might be improved.

## Methods

The wider evaluation had a longitudinal design, wherein a cohort of people who had been offered inpatient detoxification in one of the newly commissioned beds were invited to participate in three telephone interviews. The first interview occurred whilst people were waiting to start detoxification, the second occurred immediately after they left detoxification, and the third occurred twelve weeks after they left detoxification. This paper focuses on data generated during the second interviews as this is when participants were invited to talk about the inpatient detoxification staff.

Ethical approval to undertake the research was received from Glyndwr/Wrexham University, Wales (Ein Cyf 497) and recruitment occurred between March 2022 and March 2023. Professionals, based in diverse health and social care organisations across England, who were referring people to the new detoxification beds provided a study information sheet to, and discussed the study with, any individuals to whom they had offered a detoxification place. The contact details of any person who was interested in learning more about the study and was willing to speak directly to a researcher were then forwarded, with their permission, to the study team. A researcher next telephoned the interested individual to explain the study again, double-check their eligibility, and arrange the first interview.

All interviews were conducted by one of a team of qualitative researchers who had participated in two earlier training sessions (and been given accompanying briefing notes) to ensure consistency of approach. Researchers took informed consent prior to each interview and used a semi-structured topic guide, tailored for each of the three interview stages. Collectively, the topic guides had been designed to address the wider study objective of evaluating the newly commissioned inpatient detoxification bedspaces. As such, they did not specifically focus on patients’ relationships with staff, but instead explored the detoxification experience from the patient perspective. Accordingly, the focus of the topic guides was on participants’ background; substance use and treatment experiences; reasons for seeking inpatient detoxification; expectations of inpatient detoxification; experiences of inpatient detoxification (from referral through discharge); and life after inpatient detoxification. Interviews lasted approximately an hour, were audio recorded, and were transcribed verbatim by a professional transcription service. On completion of each interview, participants were given £20 as thanks and allocated a unique study number.

Transcriptions were entered into the qualitative software programme MAXQDA [24] and all content relating to detoxification staff were assigned to a single code labelled ‘staff’. The coded ‘staff’ data were then exported to Microsoft Word and analysed via a process of Iterative Categorisation [25, 26]. This involved reducing all the staff data into bullet points whilst retaining the study numbers of the participants who made each point. Similar bullet points were grouped and regrouped generating themes which collectively captured participants’ views and experiences. The findings where then further reviewed to explore similarities and differences of opinion within each theme. All coding and analyses were undertaken by the first author who had not personally conducted any of the interviews. Members of the wider team then reviewed the findings to check that they corresponded with their own perceptions of the data and to advise on interpretation.

### Participants

Thirty-two people completed an initial interview, of whom 20 (analysed here) completed a second interview (see Table 1). These 20 people included 14 males and 6 females. They were aged 28–67 years and all White British or White Other. Although their demographic details were broadly consistent with those completing a first interview, 6/8 participants using opioids at their first interview were lost to follow up. This meant that nearly all participants completing a second interview (17/20) were being treated for alcohol.

**Table 1:** Participant characteristics (self-reported)

Characteristic	Participants completing a second interview *N* = 20
**Sex**	
Male Female	14 6
**Age (years)**	
Mean (range)	45 (28–67)
**Ethnicity**	
White British White English White Irish	16 3 1
**Substance being treated**	
Alcohol Opioids Ketamine	17 2 1
**Detoxification setting**	
Specialist Service General Hospital	16 4

The 20 participants had collectively detoxified in 16 different facilities (run by the National Health Service [NHS], charities, private organisations, or mixed NHS and charity partnerships) across England. These included 12 specialist detoxification services (or units) and 4 general hospitals. Sixteen participants had been treated in a specialist detoxification service (or unit) whereas 4 had been treated in a general hospital (where they had been allocated a private room or a bed on a small ward where other patients were detoxifying). Nineteen participants reported that they had successfully detoxified from the substance(s) for which they were receiving treatment whilst one (treated in a general hospital) said he had relapsed to alcohol.

## Results

Analyses of participants’ accounts of the staff involved in their treatment generated three broad themes. These were labelled: (i) staff as people behind their roles; (ii) staff as providers of medical care and non-medical services; and (iii) staff as facilitators of treatment rules, processes and outcomes.

### Staff as people behind their roles

Participants universally reported that their treatment had involved clinical and non-clinical staff. These included doctors, nurses, support workers, healthcare assistants, counsellors, recovery workers, chefs, cleaners, administrators, managers, unpaid volunteers, students, and people who worked in other organisations but came into detoxification services to provide group work or therapy. Several participants added that paid staff and volunteers who had disclosed personal experience of AOD problems had been particularly helpful because they seemed to understand what detoxifying was like. As Participant 04 explained:

“A lot of the staff that work there have been off drugs and alcohol for like fifteen years and that, and they still struggle now… They talked about themselves as well and how they got there, and how they’ve been off it [AOD] and how they’ve struggled and how they’ve coped with it.” (Participant 04, male, specialist service).

Occasionally, participants singled out staff whom they felt had performed their job poorly, e.g., a cleaner in a general hospital who ‘slammed doors’ and ‘noisily emptied bins’ early in the morning or a service manager who was ‘rude’. More frequently, participants identified individuals whom they remembered for being particularly good at their role. These included chefs who ‘did a fantastic job’ preparing food every day for large numbers of people and staff who went ‘beyond what could have been expected to care’. Elaborating on this, Participant 03 described how one nurse had successfully dissuaded her from ending her detoxification prematurely:

“The alcohol nurse… she was absolutely amazing. She had actually finished her shift… but she… came back onto the ward… I don’t know what it was that she said made me change my mind [about self-discharging]… I said, ‘Right okay, I’ll go back’… It was thanks to her [that detoxification was completed].” (Participant 03, female, general hospital).

Participants’ most common assessment of staff was that they were ‘brilliant’, ‘amazing’, ‘fantastic’, ‘lovely’, or ‘wonderful’. In support of this, they described how staff were consistently ‘nice’ or ‘easy to get along with’ and how ‘you couldn’t find fault with them’. Two participants expressed surprise at this, noting that they had expected them to be ‘harsh’ and ‘judgemental’. Another male participant reported that he had been to the same detoxification service a few years previously and, even though many of the people working there had since changed, the staff were still ‘amazing’:

“I think there were only three members of staff that were there four years ago that were still there now. All of the staff there are amazing.” (Participant 12, male, specialist service).

Reflecting this, several participants spontaneously volunteered that people working in the service were the ‘best part’ of their treatment. In contrast, only Participant 22 (who had completed his detoxification in a general hospital) identified staff as the worst part. Whilst a few others said they disliked staff, they again usually referred to specific individuals or events and emphasised that their criticism did not extend to all staff. As Participant 28 clarified:

“I got on really well with all of the staff apart from the manager… But apart from her, the rest of the staff were absolutely amazing.” (Participant 28, female, specialist service).

When asked to describe why staff were so ‘brilliant’ or ‘wonderful’, participants identified a range of personal characteristics or qualities. These included staff being friendly, kind, helpful, caring, approachable, calm, attentive and non-judgemental. One female participant who was detoxing in a general hospital whilst pregnant said that she had felt very vulnerable, but the staff would give her a hug or hold her hand to help calm her down. Meanwhile, another described how staff would let her sit with them when she was feeling upset:

“You are detoxing, so your emotions are all over the place. When I was having a very emotional moment, the staff would let me go and sit with them in the nurses’ station.” (Participant 20, female, specialist service).

Other participants similarly reported that staff had been reassuring or had comforted them when they had felt frightened or anxious. In addition, some stated that staff liked to have a ‘laugh with you’ to ‘keep your spirits up’. Again, only Participant 22 (treated in a general hospital and introduced above) stated that he had found some of the staff to be lacking in empathy and compassion, which he attributed to them not understanding alcoholism and detoxing and so needing more education. In response to the question ‘what could have been better?’, he replied:

“Like I said, the staff. A bit more empathy, a bit more compassion, a bit of understanding, a bit of education of… what I’m going through.” (Participant 22, male, general hospital).

### Staff as providers of medical care and non-medical services

Further to their personal qualities, participants generally spoke very positively about the medical care staff had provided. For example, participants said that staff regularly checked to make sure they were feeling alright and/or gave them a buzzer to press if they felt unwell. A few explained how their medication had been changed quickly and efficiently when there were problems, and others stated that staff had been helpful in bringing their discharge forward if their treatment had progressed more quickly than expected. Additionally, participants often commented on how clearly staff had explained treatment-related issues to them. This included details of the detoxification they would be receiving, any personal health problems they were experiencing, and discharge plans. Participant 02 described the explanations he had received as follows:

“And as regards comfort and as regards how I was treated and how things were explained to me, it was 100%.” (Participant 02, male, general hospital).

Despite this, a few participants reported being unhappy about the medical care provided by staff. Participant 20 felt that aspects of her treatment had not been clearly communicated with her and, because of this, she had missed the opportunity of moving from the detoxification service into a rehabilitation programme (although she emphasised that she did not want to be critical of the staff because ‘overall they were superb’). Participant 30 complained that his diazepam had been terminated prematurely during detoxification in a general hospital, and this had resulted in him discharging himself and immediately drinking. Lastly, Participant 14 was angry that staff in the detoxification service he had attended did not notice that other patients were using substances and were slow to respond when they eventually realised what was happening:

“They [other patients] were all fucking drugged out of their head. I put a big complaint into [name of service]… It took me ten minutes to find out what these people were doing, and it took the people that worked there ten days to find out… Basically, I didn’t go to sleep for two weeks because I was frightened.” (Participant 14, male, specialist service).

Participants treated in specialist services also often praised staff for the non-medical, day-to-day services provided during detoxification. These included laundering clothes, changing bedding, offering snacks, helping with online shopping, cleaning rooms, washing plates and dishes, and providing entertainment in the form of quizzes and groups. Several participants added that when you are feeling unwell from detoxing, it means a lot to have someone helping you with domestic chores and bringing you food. As Participant 21 reflected:

“Your dinner plates and stuff all got washed up for you. All the cleaning was done, your washing was done for you. They made it as easy as they could for you on that side of things, which was great to be honest with you. Because when you’re going through detox, the last thing that you really need is the menial tasks of the day… I know in real life, they’ve got to be done… But in the detox centre, it was great to have that chance not to do them.” (Participant 21, male, specialist service).

Although participants repeatedly stated that there was always a staff member around with whom they could ‘chat’, several felt that there were insufficient opportunities to talk to staff in any depth. In this regard, some participants reported that the detoxification service had not provided any formal counselling and staff were too busy to offer them emotional support, so their mental health needs had not been addressed. A few participants were surprised and disappointed at this omission, but others said that they had not expected counselling or understood that their detoxification was not long enough to address their mental health problems properly and there was little point in starting conversations that could not be completed:

“I had a key worker, and we had a couple of one-to-ones when I was in there, but she said to me… [that] if I was going on to secondary [rehabilitation service], she didn’t want to open too many cans of worms that didn’t want to then get closed before I left. So, she was not a counsellor. She was just my keyworker while I was there.” (Participant 12, male, specialist service).

### Staff as facilitators of treatment rules, processes and outcomes

In terms of the detoxification process, participants routinely stated that they valued the privacy, freedom, and choice that staff had afforded them during their treatment. For example, some described how staff had knocked on room doors before entering, invited them into a private room if they had to see a doctor or nurse, and permitted them to spend time in their own room with the door closed if they wished:

“I could close my door, or I could have the door open… My partner came up and visited me… and they’d let us sit with the door closed and have a chat. So yeah, I was allowed privacy.” (Participant 03, female, general hospital).

Participants additionally appreciated it when staff had allowed them to stay in their rooms because they were feeling unwell, use their phones to speak to people external to the service, watch television, listen to the radio, or go to the kitchen during the night. Additionally, participants liked being able to make their own decisions about which groups and activities to join, whether to smoke tobacco, and where, when and what to eat. Indeed, participants were overall generally happy that staff had been flexible, had not adhered rigidly to rules or regulations, and had treated them ‘like adults’. Despite this, several also made it clear that they accepted that some rules were necessary and were pleased when staff were quick to sanction any substance use on the premises. As Participant 08 acknowledged: “I signed the contract, so I was aware of the rules.”

From participants’ accounts, it was evident that their interactions with staff took on extra significance at two key transition points in the treatment journey. These were on the day they began and the day they finished their detoxification. On starting their treatment, participants expressed gratitude towards staff who had welcomed them warmly, showed them round, helped them to settle in, introduced them to people, and generally given them some time to ‘find their feet’. For example, Participant 13 described how she had arrived intoxicated and was thankful that staff had let her sleep and ‘sober up’ before explaining the treatment to her:

“They [the staff] were just so supportive and caring and everything… I was taken to [name of service], but I was quite drunk because I knew that it was going to be my last drink. So, I obviously can’t really remember a lot of it, but I was just taken to my bedroom, and obviously I just went to sleep… When I woke up and I came too [staff explained everything], because they couldn’t have explained anything to me in the state that I was in.” (Participant 13, female, specialist service).

On the last day of treatment, several participants spoke warmly of how staff had given them a hug, issued them with a certificate for successfully completing their detoxification, cheered them or rang a bell in their honour, and/or helped them to a taxi with their bags. More negatively, two participants felt that staff could have done more to make the process of moving in less bewildering and anxiety-provoking and three felt that their discharge had been unsatisfactory. In this regard, one said he had been given no information about what to do when he got home; a second complained that he had not understood why he had had to leave so quickly; and a third stated he was sent home whilst craving alcohol.

Participants often commented on how they had been affected by their interactions with the staff providing their detoxification. A few, such as Participant 08, stated that they had been sad to leave and wished they could have stayed longer because of the positive bonds they had formed with staff and with other patients:

“I was a bit sad the night before being picked up. Because you become close and start seeing them [staff and other patients] as family. I know it’s only ten days, but they’re ten long days together.” (Participant 08, female, specialist service).

Mitigating this sadness, a small number of participants reported that they were pleased that staff had offered them the opportunity to visit the service in the future to give a talk or to join online meetings for ongoing support. Others expressed gratitude for the help detoxification staff had given them, noting how this had enabled them to become abstinent and/or to achieve benefits in other aspects of their lives, such as their diet, sleep, relationships, or daily routines. This included two male participants who both described how staff had helped them to manage their diabetes better, which had in turn resulted in an improvement in their physical health:

“My diet is better. My management of diabetes is better… That all started in the detox. I’ve worked hard on that for the two weeks and got a lot of support. So, I’m taking my meds [medication] at the right time. My glucose levels are okay, which they were bad before I went in. So, my physical health is better.” (Participant 25, male, specialist service).

## Discussion

Our analyses revealed a high level of positive regard for, and appreciation of, staff and the care they provided. This seems to be a departure from previous research where accounts of staff in inpatient detoxification services, specialist addiction services, and more generic treatment services have tended to be mixed [8, 13, 14]. Participants’ accounts of what they appreciated about the people working in inpatient detoxification services were, however, consistent with the existing literature. Thus, patients liked staff who were friendly, kind, helpful, caring, approachable, calm, attentive and non-judgemental [8, 11–15]. Equally, they valued staff who had personal experience of substance use [4, 8, 9], were flexible, afforded them privacy, and allowed them to make choices and be independent [8, 9, 27].

A small number of participants articulated surprise that people working in inpatient detoxification services had not been harsh or judgemental towards them. In addition, they appreciated the provision of even relatively basic non-medical care (such as laundering clothes and changing bedding). Such reactions potentially reflect low expectations related to the stigma and hostility that people who use addiction services frequently experience, including from professionals [28–30]. Participants were also grateful when staff explained aspects of the detoxification process to them and did this sensitively and at times when they were able to absorb this information, rather than when they were intoxicated or had only just arrived at the service and were feeling bewildered and vulnerable. These findings reflect staff compassion and common sense whilst also resonating with research that has shown how professionals with good communication skills have better patient outcomes than those who communicate poorly [7–10, 31].

In contrast, participants disliked staff whom they perceived to be rude, lacking in empathy or compassion, or uninformed about substance use [8, 12, 16]. They were additionally critical when they believed that staff had not communicated important information, had made unhelpful changes to their medication, or had not responded quickly to keep them safe when other patients were using substances. Lack of information, particularly when moving into and out of detoxification services, was also occasionally identified as a problem. Our findings suggested that patients potentially report more negative experiences when detoxing in general hospitals compared to specialist services. This may be because staff working in general hospitals tend to have less specialised knowledge and skills than staff working in substance use services [13, 14] and/or because general hospitals have less operational flexibility and cannot offer the same level of non-medical support as specialist services. Overall, however, criticisms of staff in all settings were relatively uncommon and largely directed at specific people and events. Indeed, participants were often keen to emphasise that, despite any adverse experiences during their detoxification, they viewed staff very positively.

Taking all our findings together, two broad concepts appeared to underpin how participants described and evaluated staff. These were ‘holistic care’ and ‘patient-centred care’. Holistic care refers to caring for the whole person [32, 33]; here, not only the patient’s substance use, but also their broader health and wellbeing. In this regard, participants recognised that their treatment comprised both clinical and non-clinical services delivered by a multi-disciplinary team. Alongside medical care, this included the day-to-day support they received, such as food, housekeeping services, and groups and activities [16]. In addition, some wanted more one-to-one time with staff to discuss how they were feeling [8, 9]. Transitions into and out of detoxification (when patients’ lives in the community interconnected with their inpatient treatment) were also identified as times when relationships with staff gained heightened relevance.

Patient-centred care, meanwhile, refers to respecting a patient’s values, preferences and expressed needs; co-ordinating and integrating care; providing information, communication, and education; ensuring the patient’s physical comfort; providing the patient with emotional support and relieving fear and anxiety; and involving family and friends to provide additional support [35, 36]. The analyses we presented did not consider patients’ relationships with family and friends in any detail, although some patients stated that they appreciated having access to their mobile phone to speak with people external to the detoxification service. Otherwise, staff demonstrated, and participants valued, all key aspects of patient-centred care. This indicates that staff delivering detoxification routinely worked in patient-centred ways and the concept of patient-centredness underpinned participants’ positive assessments of staff [9].

In recent years, there has been tendency to use the term ‘person-centred’ rather than ‘patient-centred’ care. However, research has identified some important but subtle differences between these two concepts [37]. Whilst the goal of patient-centred care is generally to help the patient establish a functional life in the present moment, the goal of person-centred care is to enable them to achieve a meaningful life in wider terms [37]. Building on this, we suggest that staff in our study tended to provide patient-centred care (focusing on how they could support patients during their time in the inpatient detoxification facility), whereas patients were often seeking person-centred care (focusing on how inpatient detoxification could improve their lives as a whole). Some dissatisfaction may therefore have occurred when staff did not, or could not, deliver the broader person-centred care that patients wanted.

These findings highlight potential tensions and dilemmas for those working in detoxification facilities. For example, staff who go beyond their professional role by sharing details of their personal life (including their own substance use), building friendships with patients who lack social networks, or providing support not directly related to detoxification may inadvertently undermine their patients’ independence by creating a ‘learned passivity’ [38]. Indeed, evidence of patients’ sadness at the prospect of leaving inpatient detoxification treatment highlights the risk of co-dependence. Relationships between patients and detoxification staff need to be carefully nurtured on arrival and not abruptly severed at the point of return to the community. Nonetheless, staff also need to help patients maintain any pre-existing positive community relationships during their treatment and build new supportive social networks after discharge [38, 39]. Given the time-limited nature of inpatient detoxification, this is likely to be facilitated if staff from community treatment services stay in touch regularly with their clients whilst they are in inpatient treatment, so improving discharge planning and continuity of care [8, 9, 34].

Returning to the concept of the therapeutic alliance, patients may, of course, feel positive about staff whilst staff do not reciprocate this feeling [4]. Since our research did not ascertain staff views of their patients, we cannot generate definitive conclusions about the therapeutic alliance between patients and inpatient detoxification staff. However, it seems reasonable to assume that patients who like staff and feel that they are helping them will be more likely to develop a productive therapeutic relationship with them [4–6]. Reflecting this, some of our participants described how staff had helped them to become abstinent, improve their health, and achieve wider benefits related to recovery, such as better daily routines [40–42]. Accordingly, our data seem to confirm that holistic and patient-centred care both increase the therapeutic alliance and lead to better treatment outcomes. Moreover, the therapeutic alliance is not limited to relationships between patients and healthcare professionals; it was also evident between patients and non-clinical staff within inpatient detoxification settings.

### Limitations

As with any research, the results presented have limitations. Most obviously, they derive from one qualitative study conducted in England during a period of investment in inpatient detoxification. Thirty-two people were recruited to the research whilst they were waiting for their detoxification to start but only 20 were re-interviewed after their detoxification. Of these, 19 reported that they had successfully completed their treatment. Those who were recruited were all White and most were male and being treated for alcohol. This raises questions about who does not engage in research and who does not have access to inpatient detoxification, even during a period of treatment expansion. Additionally, there is no record of what happened to the twelve people who were not reinterviewed. This is particularly relevant since a disproportionate number of people using opioids were lost to follow up, and their accounts of staff may have been less positive than those described.

## Conclusions

The aim of this paper was to analyse patients’ accounts of inpatient detoxification staff to ascertain whether, and if so how, relationships with them, and potentially the therapeutic alliance, might be improved. Patients appreciated both clinical and non-clinical staff, and considered staff qualities and characteristics, how staff behaved towards them, the medical and wider services staff provided, points in the treatment pathway when relationships with staff were particularly important, and the impact staff had had on them personally. Patients’ accounts of staff were predominantly positive, and their criticisms were generally limited to specific individuals or events. Despite this, there was scope for improvement in some services. Rudeness, indifference, and lack of empathy should not feature in any treatment pathway; good communication, opportunities for emotional support, and heightened vigilance to prevent non-prescribed substance use that might compromise patient safety always need to be considered; and all staff who have contact with detoxifying patients need appropriate knowledge and training, including how to manage professional boundaries. In conclusion, the largely positive accounts of staff that we identified may not be representative of all inpatient detoxification services in the UK. Nonetheless, the holistic and patient-centred approach to working that most staff in our study adopted appears to contribute to a good therapeutic alliance.

## Data Availability

The dataset generated and analysed during the current study is not publicly available due to small sample size, sensitive data, and potential identification of organisations and individuals contra confidentiality agreements; please contact the corresponding author for further information.
